# Integrative analysis of microbiome and metabolome revealed the effect of microbial inoculant on microbial community diversity and function in rhizospheric soil under tobacco monoculture

**DOI:** 10.1128/spectrum.04046-23

**Published:** 2024-07-11

**Authors:** Xianjun Lai, Wangjun Duan, Wenyou Zhang, Zhengsong Peng, Xianjun Wang, Haiyan Wang, Xiaobo Qi, Huaqiang Pi, Kailu Chen, Lang Yan

**Affiliations:** 1 Panxi Crops Research and Utilization Key Laboratory of Sichuan Province, Xichang University, Liangshan, China; 2 China Tobacco Sichuan Industrial Co. Ltd, Chengdu, China; 3 Sichuan Key Laboratory of Molecular Biology and Biotechnology, College of Life Sciences, Sichuan University, Chengdu, China; Agroscope, Nyon, Switzerland

**Keywords:** tobacco, continuous monocropping, microbial inoculant, microbial diversity, metabolite spectrum

## Abstract

Over-application of chemical fertilizers and continuous cropping obstacles seriously restrict the sustainable development of tobacco production. Localized fertilization of beneficial microbes has potential advantages in achieving higher productivity, but the underlying biological mechanisms of interactions between rhizospheric microorganisms and the related metabolic cycle remain poorly characterized. Here, an integrative analysis of microbiomes with non-targeted metabolomics was performed on 30 soil samples of rhizosphere, root surrounding, and bulk soils from flue‐cured tobacco under continuous and non-continuous monocropping systems. The analysis was conducted using UPLC-MS/MS platforms and high-throughput amplicon sequencing targeting the bacterial 16S rRNA gene and fungal ITS gene. The microbial inoculant consisted of *Bacillus subtilis*, *B. velezensis,* and *B. licheniformis* at the ratio of 1:1:1 in effective microbial counts, improved the cured leaf yield and disease resistance of tobacco, and enhanced nicotine and nitrogen contents of tobacco leaves. The bacterial taxa *Rhizobium*, *Pseudomonas*, *Sphingomonadaceae*, and *Burkholderiaceae* of the phylum *Proteobacteria* accumulated in high relative abundance and were identified as biomarkers following the application of the microbial inoculant. Under continuous monocropping, metabolomics demonstrated that the application of the microbial inoculant significantly affected the soil metabolite spectrum, and the differential metabolites were significantly enriched to the synthesis and degradation of nicotine (nicotinate and nicotinamide metabolism and biosynthesis of alkaloids derived from nicotinic acid). In addition, microbes were closely related to the accumulation of metabolites through correlation analysis. The interactions between plant roots and rhizospheric microorganisms provide valuable information for understanding how these beneficial microbes affect complex biological processes and the adaption capacity of plants to environments.

**IMPORTANCE:**

This study elaborated on how the microbial fertilizer significantly changed overall community structures and metabolite spectrum of rhizospheric microbes, which provide insights into the process of rhizosphere microbial remolding in response to continuous monocropping. we verified the hypothesis that the application of the microbial inoculant in continuous cropping would lead to the selection of distinct microbiota communities by establishing models to correlate biomarkers. Through correlation analysis of the microbiome and metabolome, we proved that rhizospheric microbes were closely related to the accumulation of metabolites, including the synthesis and degradation of nicotine. The interactions between plant roots and rhizospheric microorganisms provide valuable information for understanding how these beneficial microbes affect complex biological processes and the adaption capacity of plants to environments.

## INTRODUCTION

The application of chemical fertilizers has significantly increased crop production by 30%–50% owing to the promotion of plant growth through increasing plant nutrition and changing soil fertility (1). However, in recent decades, the overuse of chemical fertilizers for increased crop production resulted in deficiencies of soil organic carbon owing to excessive supplementation of inorganic nitrogen, which disturbed the balance of soil microbial communities through the recruitment of fewer beneficial bacteria and more pathogens in the rhizosphere soil of crops (2). Tobacco production has experienced the overuse of chemical fertilizers for a long time in the pursuit of high yield, along with increasingly obvious continuous cropping obstacles of flue-cured tobacco. This has triggered a series of degradation problems of tobacco-planting soil, such as salinization, hardening, microbial activity decline, and decreased utilization rate of soil nutrients (3). Continuous cropping obstacles are a major problem in flue-cured tobacco production, resulting in a serious reduction of crop biomass and quality and increased susceptibility to crop disease (4). Although no consistent conclusion is available regarding the underlying mechanism of continuous cropping obstacles, a few factors related to the soil ecosystem have been proposed, such as imbalance of soil nutrients, autotoxic effect, and disruption of the soil microbial community (3, 5). Crops and soil microbes are inextricably linked and mutually influence one another. Microorganisms play a crucial role in soil nutrient transformation, soil health, and sustainability of soil productivity (6). Continuous cropping of tobacco was found to affect the communities of bacteria and fungi in the soil to different extents (4, 5). Furthermore, we previously reported that bacterial community diversity in the tobacco rhizosphere decreased with fewer interactions among microbial communities as the duration of continuous monocropping increased (7). In addition, studies found that the major microbial physiological groups in rhizosphere soils correlate with the intrinsic quality of tobacco, in which the contents of reducing sugar, total nitrogen, nicotine, chlorine, and potassium in tobacco are important manifestations of tobacco quality improvement (8).

Appropriate fertilization could optimize the structure of microbial communities in the rhizosphere soil of tobacco in continuous cropping systems, thereby improving microbial diversity and alleviating continuous cropping obstacles. In recent years, biofertilizers such as microbial and biochar fertilizers have been widely used in Chinese tobacco fields and proven to enhance the production and intrinsic quality of tobacco leaves (3, 9). Microbial fertilizers have stable and enduring fertilizer efficiency and have potential application value in reshaping rhizospheric microbial diversity and strengthening the fertility of tobacco-planting soil, which helps the growth of vital microorganisms in rhizosphere soil, namely plant growth-promoting rhizobacteria (PGPR) (10). PGPR encompass rhizobacteria that form specific symbiotic relationships with plants, synthesize specific compounds for plants, and capture or mobilize nutritionally important plant nutrients from non-usable to usable forms through biological processes. For example, phosphate- and potassium-solubilizing bacteria, such as *Bacillus megaterium* and *B. mucilaginosus*, may enhance mineral uptake by plants through solubilizing insoluble P and releasing K from silicate in soil (11). Another lucrative property of PGPR is to elicit the defense system and suppress the incidence of soil-borne diseases through versatile mechanisms of niche exclusion, direct antagonism, colonization, and the production of several secondary metabolites (12). The applicability of PGPR—including species of the genera *Acinetobacter*, *Bacillus*, *Enterobacter*, *Lysobacter*, and *Pseudomonas*—as biological control agents has been evaluated (13). *Bacillus* spp. can promote the growth of tobacco plants and are widely used as biocontrol agents against soil-borne diseases owing to their ability to produce broad-spectrum antimicrobial compounds and successfully colonize the host rhizosphere (14–16).

The use of PGPR microorganisms as microbial fertilizers, more appropriately called microbial inoculants, has the potential to improve tobacco production and nutrient availability as well as overcome the continuous cropping obstacles. However, the application of microbial inoculants has not achieved constant effects. The mechanisms and interactions among these microbes are still poorly understood, especially in real applications. The predominant reason for the failure of exogenous microbial inoculants is that microbial interactions between the inoculated microbes (extrinsic) and the native microbes (intrinsic) lead to incomplete structure and function of microbial communities (17). Microbial interactions—including symbiotic, synergistic, predation, parasitic, and competitive interactions—can greatly affect the diversity, structural stability, and metabolic activity of the microbial communities (18). Therefore, it is vital to conduct rational addition of exogenous microbial inoculants to activate interactions between microorganisms and strengthen the metabolic capacity of the microbial community, based on detailed studies on the addition of microbes and their effects on the structure and function of complete microbial communities. In recent years, numerous studies have been conducted to understand the characteristics of rhizospheric microbial communities in different crops or soil types, and the effects of different fertilizers on rhizospheric microbial diversity were reported in monoculture tobacco (13, 14, 17). However, the quantitative changes of microorganisms in rhizospheric soils were emphasized, while the functional diversity and the metabolic capacity of microbial communities and the correlation with tobacco production characteristics and intrinsic quality were seldom mentioned.

This study, through the integrative analysis of microbiome and metabolome, explored the effects of compound microbial inoculants on agronomic traits, disease resistance, and intrinsic quality in tobacco production, as well as the functional diversity and metabolite characteristics of rhizospheric microbial communities in a field with 20-year continuous cropping of tobacco. The objectives of this study were as follows: (i) prove that the application of suitable microbial inoculants could reduce rates of inorganic fertilizer and improve tobacco production; (ii) determine the key biomarkers in response to microbial inoculants, and the relationship between these phylotypes and tobacco index; and (iii) elaborate the interactions among the microorganisms and the metabolic capacity in the rhizospheric microbial communities. Findings from the study were expected to facilitate the understanding of the mechanism of continuous cropping obstacles and provide feasible strategies for alleviating this problem in tobacco production.

## MATERIALS AND METHODS

### Tobacco plant growth and sampling

Field experiments were conducted in a commercially long-term cultivated tobacco field from 2020 to 2022, the standardized raw material planting base of flue-cured tobacco named “Kuan&Zhai Garden” established by China Tobacco Sichuan Industrial Co., Ltd. This site is located at Wudongde town, Huidong County, Liangshan, southwest China (102°13′ E, 26°12′ N; elevation: 1874 m a.s.l.). There is a long history of flue-cured tobacco cultivation in this area, with some sites under continuous monocropping for 30 years. The soil type in the experimental fields was ACfa (Alumi-Ferric Alisols) according to the FAO-UNESCO Soil Map of the World. The fields were flat with uniform fertility in the long term. Tobacco growers apply tobacco-special compound chemical fertilizers at the rate of 750 kg ha^−1^ annually, with the total nutrient ≥42%, N:P_2_O_5_:K_2_O at 10:12:18.

The treatment group was designed with traditional compound fertilizer plus exogenous inoculant of *Bacillus* spp, which comprised *B. subtilis*, *B. velezensis,* and *B. licheniformis* at the ratio of 1:1:1 in effective microbial counts (EFU); the minimal bacterial density was 10^7^ CFU/g. Strains were self-separated, then identified and deposited in the China Center for Type Culture Collection, CCTCC, Wuhan, China (Registration No. M20231257). The control group was designed with only traditional compound fertilizer. The basal fertilizer for both groups was 50% traditional compound fertilizer, which was placed 10 cm distant from the hill and covered with 10–15 cm soil. The remaining fertilizer was applied to the control group approximately 30 days after seedling transplantation, while a microbial inoculant of 0.4 g per plant was applied to the treatment group through root irrigation and leaf spraying 7 days after seedling transplantation. The microbial inoculant was then applied once every 3 weeks and continuously used at least four times. A 3-year location experiment was performed, to avoid the soil microbiota from being disturbed by the climate environment of a certain year or by short-term fertilization disturbances. The rhizosphere soils were sampled during the tobacco harvest period in the third year.

The area of sampling was restricted to a field of treatment and control groups, which were separated into six sampling regions represented by replications. Each sample was collected from five individual plants within a field region of 110 m^2^, at distances of approximately 10 m from each other. To collect rhizosphere soil (RS), roots were first shaken to remove large clumps of soil (loosely adhering soil from roots was shaken off as surrounding soil samples, SS), then the roots with tightly adhering soil were placed into 50 mL falcon tubes containing 15 mL autoclaved phosphate-buffered saline (PBS) solution and transported on ice to the laboratory. The roots with soil attached were vortexed in PBS solution with cyclical washing in fresh PBS solution until no soil particles were visible in the solution. All the collected PBS solutions were centrifuged at 12,000 rpm for 10 min, the supernatants were removed, and 10 mL of the resuspended slurry was used for DNA extraction.

### Growth parameter and yield measurements

At the end of vegetative growth, five plants were selected randomly in each sampling region considering the border effect. To calculate the plant height before the flowering stage, the height of five plants was measured separately from the soil surface to the highest part of the plant aerial parts in centimeters. Also, the maximum length and width of the leaves were measured in each plant. Their average was recorded as the growth parameter of the region.

Generally, 3–4 tobacco leaves were harvested at a time and then cured in bulk based on a local tobacco curing method. At the end of each harvesting and curing period, the tobacco leaves of each treatment region were weighed separately, and the dry weight of tobacco was recorded as dry leaf yield for the treatment regions.

### Incidence and disease index calculation

The symptoms of tobacco bacterial disease were investigated in the field, and weatherfleck, mosaic, and wildfire of tobacco were monitored at growth (70 days after transplanting) and harvest period (118 days after transplanting). Disease incidence (I) and disease index (DI) were measured using the given formula

I = DI = [∑ (the number of bacterial wilt crops in this index × severity scale)/(total number of crops inspected × the highest severity scale)] ×100

The disease severity scale was evaluated by visual observation of the systemic leaf (fourth fully expanded leaf from the top) following a rating scale of 0 to 3, in which 0 = no symptoms; 1 = mild disease symptom sparsely distributed on leaf surface; 2 = dark-green disease symptom spread over 50% of leaf area accompanied by leaf distortion and stunting; and 3 = severe dark-green disease symptom on whole leaf, leaf distortion, leaf narrowing, and severe plant stunting.

Similarly, the control efficacy of microbial inoculants was also calculated based on DI.

Control efficacy = [(Control DI − Treatment DI)/Control DI] ×100%

### Measurement of economic traits of flue-cured tobacco leaves

The International Standard GB2635–92 was used to grade the initial flue-cured tobacco leaves, and the average price was the local price of that year. The average prices and proportions of superior-, medium-, and low-grade tobacco were recorded in different treatment groups.

### Measurement of the chemical composition of tobacco leaves

Chemical indices measurements were conducted by the Quality Supervision Department of Technical Center (Analysis and Test Center) of China Tobacco Sichuan Industrial Co. Ltd. In detail, the *Tobacco and Tobacco Products–Determination of Water Soluble Sugars–Continuous Flow Method* (YC/ T159–2002) was used to measure reducing sugar and total sugar; the *Tobacco and Tobacco Products–Determination of Total Nitrogen–Continuous Flow Method* (YC/T161–2002) was used to measure total nitrogen; the *Tobacco and Tobacco Products–Determination of Nicotine–Continuous Flow Method* (YC/T160– 2002) was used to measure nicotine; and the *Tobacco and Tobacco Products–Determination of Starch–Continuous Flow Method* (YC/T 216–2014) was used to measure starch content.

### Functional diversity of the microbial community in tobacco rhizospheric soil

Microbial DNA was isolated using the E.Z.N.A. Soil DNA Kit (Omega Bio-Tek, USA) according to the manufacturer’s instructions. DNA purity was quantified by a NanoDrop spectrophotometer and DNA quality and integrity were assessed by 1% agarose gel electrophoresis. Total DNA was amplified with bacterial 16S rRNA gene primers [338F (5′-ACTCCTACGGGAGGCAGCAG-3′) and 806R (5′-GGACTACHVGGGTWTCTAAT-3′) and fungal ITS primers [1737F (5′-GGAAGTAAAAGTCGTAACAAGG-3′) and 2043R (5′-GCTGCGTTCTTCATCGATGC-3′)], respectively, targeting the V3–V4 region and the ITS1 region. The resultant PCR products were purified, pooled in equimolar concentrations, and pair-end sequenced using the Illumina MiSeq platform (Illumina, Inc., CA, USA) at Biomarker Technologies Corporation (Beijing, China). Sequence processing, operational taxonomic unit (OTU) clustering and filtering, microbial community diversity analysis, and statistical analysis were conducted following in-house scripts and procedures in our laboratory referring to our previous study (7). Briefly, raw sequence data were quality-filtered and assembled using Trimmomatic (19) and FLASH (20), respectively. Tags containing ≥3 ambiguous N bases (chimeric sequences identified in UCHIME (21) and having a low-quality score (average Phred score of the bases ≤ 20) were further filtered, the remaining effective tags were clustered into OTUs at a similarity level of 97% using USEARCH (22). OTUs occurring in less than 5% of the samples were filtered from the OTU table. OTUs were annotated with taxonomic information using the databases SILVA 138 for bacteria or UNITE 7.2 for fungi (23).

Diversity analysis was conducted using an R script. Differential OTU abundance was performed using Wilcoxon rank sum tests based on OTUs with median relative abundance from each group >0.2%, and corresponding *P* values were corrected for multiple tests using a false discovery rate (FDR) set at 0.05. Alpha rarefaction diversity was calculated a QIIME diversity analyses workflow script core_diversity_analyses.py and unconstrained principal coordinates analysis (PCoA) was performed using the vegan capscale() function in R by specifying an intercept-only model (R Code: capscale(log_2_(RA)−1), and permutational multivariate analysis of variance (PERMANOVA) was conducted using the adonis() function from the vegan package (). All graphs and plots were generated using the ggplot2 package.

### Metabolome analysis of tobacco rhizospheric soil

To prepare samples for metabolome sequencing, 50 mg soil samples was mixed with 1,000 µL extraction solution (methanol:acetonitrile:water = 2:2:1) containing 2 µL L-2-chlorophenylalanine as an internal standard (Aladdin, China) by vortexing. Ceramic beads were added for grinding at 45 Hz for 10 min, followed by ultrasonic treatment on ice for 10 min, then stewing at −20°C for 1 h. Samples were centrifuged at 12,000 rpm for 15 min at 4°C and 500 µL supernatant was diluted with LC-MS grade water to a final concentration containing 60% methanol. The sample was transferred to a fresh Eppendorf tube with a 0.22 µm filter and then centrifuged at 12,000 rpm for 15 min at 4°C. Subsequently, 120 µL supernatant was transferred into a 2 mL injection bottle and then injected into the LC-MS/MS system (Waters UPLC Acquisition I-Class PLUS, Waters, USA) coupled with a UPLC HSS T3 column (2.1 mm×100 mm, 1.8 um, Waters) and high-resolution mass spectrometry (Waters UPLC Xevo G2-XS QTOF, Waters, USA) for analysis. The gradient elution was performed using mobile phase A and phase B. Phase A was 0.1% vol/vol formic acid aqueous solution and phase B was acetonitrile with 0.1% vol/vol formic acid in positive (pos) and negative (neg) ion mode, respectively. The Waters mass spectrometer was used to gain primary and secondary mass spectrometer data in MSe mode under the control of the acquisition software (MassLynx V4.2, Waters). In each data acquisition cycle, dual-channel data acquisition can be performed on both low collision energy (2 V) and high collision energy (10–40 V), and the scanning frequency is 0.2 seconds. The parameters of the ESI ion source were as follows: capillary voltage: 2,000 V (pos) or −1,500 V (neg); cone voltage: 30 V; ion source temperature: 150°C; desolvent gas temperature 500°C; backflush gas flow rate: 50 L/h; and desolventizing gas flow rate: 800 L/h.

The raw data collected using MassLynx V4.2 is processed by Progenesis QI V2.3 software (Nonlinear Dynamics, Newcastle, UK) for baseline filtering, peak recognition, retention time correction, and peak alignment, which produced the retention time, mass charge ratio, peak intensity, and data matrix. The main parameters of 5 ppm precursor tolerance, 10 ppm product tolerance, and 5% product ion threshold were applied. Ion peaks with a missing value (ion intensity = 0) in more than 50% of groups were deleted, as well. the zero value was replaced with half the minimum value. Compounds with resulting scores less than 36 (out of 60) were also deleted to generate a data table of substance peaks and metabolites (24). The characteristic peak was detected and the information from the MS and MS/MS analyses was mapped with metabolic-specific database; the online METLIN database (25) and self-built library at the Biomarker biotech CO., Ltd., Beijing, China, were used for metabolite identification, and at the same time, theoretical fragment identification. The identification of compounds is based on the precise mass-to-charge ratio (M/z), secondary fragments, and isotope distribution. The mass deviation of molecular ions was set to <100 PPM and deviations of fragment ions were set to <50 PPM. Finally, the metabolites were identified according to the matching scores of the secondary MS. The data were normalized with Pareto scaling and log-transformed before further analysis.

Differential metabolites were identified based on a fold change (FC) ≥ 2 or FC ≤ 0.5, the significance of the differential metabolites between the two groups (*P*-value < 0.05, *t*-test), and the variable importance in the projection (VIP) value of the orthogonal partial least-squares discriminant analysis (OPLS-DA) model (VIP >1). The Kyoto Encyclopedia of Genes and Genomes (KEGG) database (26) was used to annotate metabolites, and metaboAnalyst V5.0 (27) combined with the KEGG database was used for metabolic pathway analysis.

## RESULTS

### Effect of the microbial inoculant on tobacco growth, resistance, and economic Traits

The microbial inoculant exhibited positive and significant effects on tobacco growth and disease resistance (Table 1). Bio-inoculation of beneficial bacteria resulted in a significant increase in plant height, leaf number, and maximum leaf length. In non-continuous cropping plots, the plant height of 3.18–10.4 cm in treatment groups was higher than that in the control group and 1.38–1.64 cm higher in treatment groups were also observed in continuous cropping plots. The disease incidence and index of weather fleck, mosaic disease, and wildfire disease in treatment groups were markedly lower than those in CK groups (Table 1). In non-continuous cropping plots, the control efficacy in the microbial inoculant treatment of weather fleck and wildfire disease was 100% and that of mosaic disease was 86.43%. In continuous cropping plots, the control efficacies of the three diseases were 54.13% (weather fleck), 61.64% (mosaic disease), and 100% (wildfire disease). In addition to biometric traits in the grand growth period above, similar results were observed in the harvest period (Table S1), in which 38.41–48.69 cm and 14.35–16.75 cm higher in treatment groups versus controls were observed in non-continuous and continuous cropping plots, respectively. Furthermore, in the harvest period, the treatment groups exhibited outstanding performance on control efficacy of the three investigated diseases, with the disease index of weather fleck decreasing from 73.7% in CK to 25.13% in the treatment group in continuous cropping plots. In summary, although the addition of microbial inoculant improved the growth and disease resistance of tobacco more significantly in non-continuous cropping plots, it also increased the growth parameter and decreased the disease index in continuous cropping soil.

**TABLE 1:** Effect of microbial inoculants on growth parameters and resistance of tobacco in growth periods^*a*^

		Agronomic traits				Disease index (%)		
		Plant height (cm)	Leaf number	Maximum leaf length (cm)	Maximum leaf width (cm)	Weather fleck	Mosaic disease	Wildfire disease

Non-CC	Treatment group	108.80 ± 5.89	19.41 ± 1.66	76.78 ± 3.25	26.28 ± 2.15	0.00 ± 0.00	2.23 ± 1.53	0.00 ± 0.00
	Control group	102.01 ± 9.50	19.07 ± 1.57	76.67 ± 5.28	28.70 ± 5.19	4.33 ± 1.15	16.43 ± 1.83	1.51 ± 0.44
CC	Treatment group	102.72 ± 4.99	19.45 ± 1.53	75.64 ± 2.32	26.68 ± 3.37	3.67 ± 0.58	7.20 ± 1.20	0.00 ± 0.00
	Control group	101.21 ± 5.12	19.42 ± 1.81	75.11 ± 2.78	26.82 ± 1.68	8.00 ± 1.00	18.77 ± 1.58	2.13 ± 0.60

^*a*^ Non-CC: non-continuous cropping; CC: continuous cropping.

Significant increases in the dry matter of cured leaf yield were observed when microbial inoculant was applied (Table 2). The maximum yield of cured leaves was 150.4 kg/667 m^2^ in non-continuous cropping plots, while the yield was even higher in continuous cropping plots, reaching 151.3 kg/667 m^2^. The increase of cured leaf yield in treatment plots resulted in the output values of the plots increasing by 1256.85 and 1286.93 CNY/667 m^2^ for non-continuous and continuous cropping plots, respectively. In addition, the grade index of cured leaf showed that, compared with CK groups, the proportion of superior grade cured leaf increased by 2.8% and 1.14% with inoculation, and that of low grade decreased by 1.2% and 0.3%, in non-continuous and continuous cropping plots, respectively.

**TABLE 2:** Effect of microbial inoculants on yield and economic trait of tobacco leaf^*a*^

		Proportion in grading (%)			Dry yield (kg/667 m^2^)	Output values (CNY/667 m^2^)	Average price (CNY/kg)
		Superior grade	Medium grade	Low grade			
Non-CC	Treatment group	74	23.87	2.13	150.4	5265.16	35.01
	Control group	73.23	24.72	2.05	117	4008.31	34.26
CC	Treatment group	71.2	26.32	2.48	151.3	5258.62	34.76
	Control group	73.83	23.82	2.35	115	3971.69	34.54

^*a*^ The grading of flue-cured tobacco leaves is based on the international standard of “GB2635-92.”

### Effect of the microbial inoculant on tobacco intrinsic quality

The chemical composition of tobacco leaves determines their intrinsic quality, in which a valuable quality assessment criterion in tobacco is nicotine content. As shown in Table 3, the microbial inoculant promoted leaf nicotine content. In the treatment groups, nicotine content ranges from 2.0–3.0% to 1.5–2.0% were recorded in middle- and down-stalk tobacco leaves, respectively, in both non-continuous and continuous cropping plots. However, the nicotine contents in the CK groups were either low or high in comparison. The increases in nicotine content were attributed to the positive effects of microbial inoculant application on nitrogen content in tobacco leaves (see Discussion for details). Microbial inoculant application had a significantly positive effect on the nitrogen content of tobacco leaves as evidenced by the observed increase in nitrogen content in upper and middle leaves. Although the nitrogen contents in down-stalk tobacco leaves were decreased in treatment groups compared with that in CK groups, the contents were closer to the standard scope (1.3–1.8% in down-stalk tobacco leaves). There was also a positive and significant effect of microbial inoculant on the contents of total sugar and reducing sugar, which are important indexes in the tobacco industry. Sugar contents in treatment groups were significantly increased compared with those of CK groups, although they exceeded the standard scope in upper and middle leaves in both groups. The increase in sugar contents caused by microbial inoculant application benefited down-stalk tobacco leaves, which had sugar contents closer to the standard scope in treatment groups. The ratio of reducing sugars and nicotine contents (S/Nic) is often used to describe the concordance of the tobacco compounds. Although S/Nic in upper and middle leaves did not vary significantly between treatment and CK groups, the down-stalk tobacco leaves had a more suitable S/Nic in the treatment groups than in the CK groups, demonstrating a significant improvement following microbial inoculant application.

**TABLE 3:** Effect of microbial inoculants on chemical constituents and intrinsic quality of tobacco leaves^*a*^

			Nicotine %	Reducing sugar %	Total sugar %	Total nitrogen %	pH	Starch %	TS-RS	N/Nic	S/Nic
Upper leaves (B3F)	Non-CC	Treatment group	3.92 ± 0.25	24.2 ± 2.53	28.7 ± 2.49	2.41 ± 0.29	5.78 ± 0.14	4.66 ± 0.37	4.5 ± 0.04	0.61 ± 0.03	6.17 ± 0.25
		Control group	2.94 ± 0.23	22.4 ± 2.48	26.8 ± 2.83	2.1 ± 0.26	5.81 ± 0.11	4.42 ± 0.43	4.4 ± 0.35	0.71 ± 0.03	7.62 ± 0.25
	CC	Treatment group	3.85 ± 0.19	26.2 ± 3.16	28.2 ± 2.58	2.52 ± 0.17	5.72 ± 0.17	4.27 ± 0.39	2.0 ± 0.58	0.65 ± 0.01	6.81 ± 0.49
		Control group	3.82 ± 0.38	22.9 ± 2.69	26.4 ± 3.18	2.48 ± 0.21	5.72 ± 0.18	4.73 ± 0.33	3.5 ± 0.49	0.65 ± 0.01	5.99 ± 0.11
Middle leaves (C3F)	Non-CC	Treatment group	2.53 ± 0.38	26.0 ± 3.58	29.1 ± 3.59	1.79 ± 0.21	5.85 ± 0.12	1.66 ± 0.38	3.1 ± 0.01	0.71 ± 0.05	10.28 ± 0.13
		Control group	1.94 ± 0.29	17.8 ± 2.19	23.0 ± 2.84	1.52 ± 0.14	6.00 ± 0.11	1.05 ± 0.25	5.2 ± 0.65	0.78 ± 0.03	9.18 ± 0.25
	CC	Treatment group	2.20 ± 0.31	25.9 ± 3.28	29.3 ± 3.32	1.72 ± 0.18	5.71 ± 0.17	1.25 ± 0.22	3.4 ± 0.04	0.78 ± 0.06	11.77 ± 0.17
		Control group	1.78 ± 0.28	18.7 ± 1.99	22.7 ± 2.94	1.57 ± 0.11	5.20 ± 0.12	1.94 ± 0.29	4 ± 0.95	0.88 ± 0.06	10.51 ± 0.55
Down-stalk tobacco (X2F)	Non-CC	Treatment group	1.66 ± 0.11	15.9 ± 2.59	18.8 ± 2.75	1.96 ± 0.14	5.94 ± 0.13	1.05 ± 0.19	2.9 ± 0.16	1.18 ± 0.03	9.58 ± 0.93
		Control group	2.42 ± 0.36	7.20 ± 1.59	10.9 ± 1.49	2.58 ± 0.18	5.52 ± 0.13	1.05 ± 0.23	3.7 ± 0.10	1.07 ± 0.07	2.98 ± 0.22
	CC	Treatment group	2.09 ± 0.19	14.8 ± 1.93	16.9 ± 1.92	1.93 ± 0.21	6.02 ± 0.18	1.05 ± 0.24	2.1 ± 0.01	0.92 ± 0.01	7.08 ± 0.28
		Control group	2.75 ± 0.24	7.20 ± 1.79	13 ± 1.88	2.6 ± 0.26	5.64 ± 0.11	2.28 ± 0.22	5.8 ± 0.09	0.95 ± 0.01	2.62 ± 0.43

^*a*^ TS-RS: differences between total and reducing sugars; N/Nic: the ratio of nitrogen and nicotine; S/N: the ratio of sugars and nicotine.

### Microbial community structure variations in response to microbial inoculant

Bacterial and fungal community profiles were generated based on 30 samples collected from the bulk soil, rhizosphere soil, and root-surrounding soil of continuous cropping and non-continuous cropping plots, with groups of microbial inoculants and CK, respectively. Through amplification and sequencing of the V3–V4 region of the 16S rRNA gene and ITS1 region, 2,394,918 bacterial effective tags (average, 79,831 clean tags; range, 79,291–80,179 clean tags per sample) and 2,375,748 fungal effective tags (average, 79,192 clean tags; range, 64,028–80,233 clean tags per sample) were obtained after quality control.

**Fig 1:**
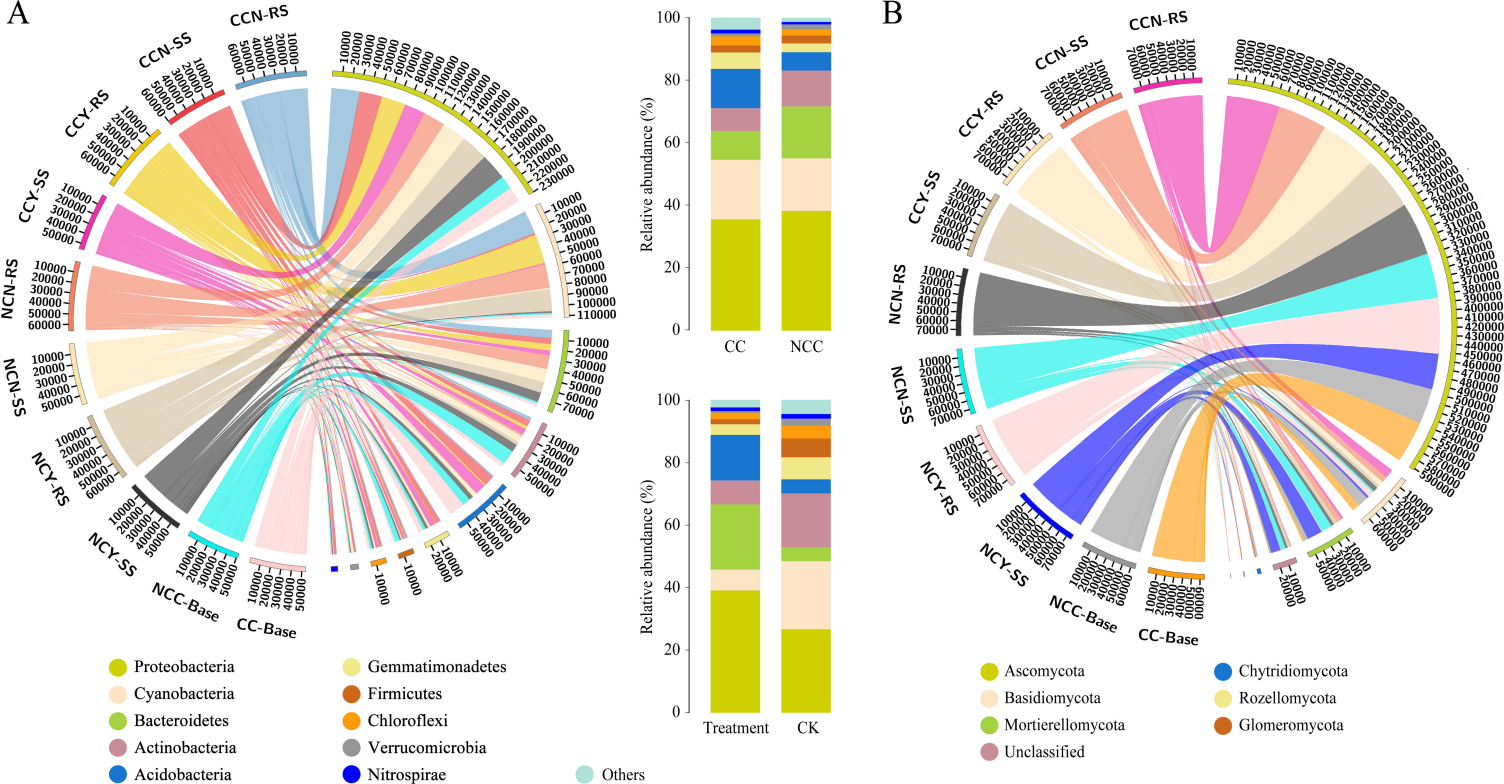
Relative abundances of dominant bacterial (**A**) and fungal (**B**) phyla. CCY: Continuous cropping plots with microbial inoculants applied; CCN: continuous cropping plots without microbial inoculants applied; NCY: non-continuous cropping plots with microbial inoculants applied; NCN: non-continuous cropping plots without microbial inoculants applied; RS: rhizosphere soils; SS: root-surrounding soils.

For the bacterial community, clean tags were clustered into 1,353 OTUs at 97% sequence similarity, represented by different bacterial species belonging to 23 phyla, 66 classes, 137 orders, 219 families, and 399 genera. At the phylum level, the top 10 dominant phyla in terms of relative abundance were *Proteobacteria*, *Cyanobacteria*, *Bacteroidetes*, *Acidobacteria*, *Actinobacteria*, *Gemmatimonadetes*, *Firmicutes*, *Chloroflexi*, *Verrucomicrobia*, and *Nitrospirae*, representing more than 90% of all sequences. Phylum abundances of the bacterial community across different samples are shown in Fig. 1A. There was an obvious difference in bacterial community abundances between rhizosphere and root-surrounding soils. In rhizosphere soils, *Proteobacteria*, *Cyanobacteria*, and *Bacteroidetes* accounted for more than 80% of the bacteriome, while *Acidobacteria*, *Actinobacteria*, and *Gemmatimonadetes* were recorded in high abundances in the root-surrounding soils; in particular, *Acidobacteria* had relatively high abundances in continuous-cropping root-surrounding soils. Significant differences were observed in the relative abundances of the top 10 phyla in different groups (Fig. 1A). After grouping the samples based on the cropping system, the relative abundances of *Acidobacteria* and *Gemmatimonadetes* increased while those of *Cyanobacteria*, *Bacteroidetes*, and *Actinobacteria* decreased in continuous cropping samples compared with non-continuous cropping samples. Similarly, compared with CK groups, the treatment groups exhibited reduced relative abundances of *Acidobacteria*, *Actinobacteria*, *Gemmatimonadetes*, and *Firmicutes*, but increased relative abundance of *Proteobacteria*, *Cyanobacteria*, and *Bacteroidetes*.

The fungal communities consisted of 616 OTUs clustered at 97% sequence similarity. The dominant phyla across all samples were Ascomycota, Basidiomycota, Mortierellomycota, Chytridiomycota, Rozellomycota, and Glomeromycota (Fig. 1B). Quantitatively, fungi of the phylum Ascomycota were present in large quantities (>80% relative abundance), especially in rhizosphere soils. The species composition of rhizosphere soil was simpler than that of root-surrounding and bulk soils, with only Ascomycota, Basidiomycota, and Mortierellomycota species in continuous cropping samples and more unclassified species in non-continuous cropping samples. In addition, microbial inoculant treatment had little effect on fungal community composition compared with the control (CK) treatment.

### Microbial diversity influenced by microbial inoculant

**Fig 2:**
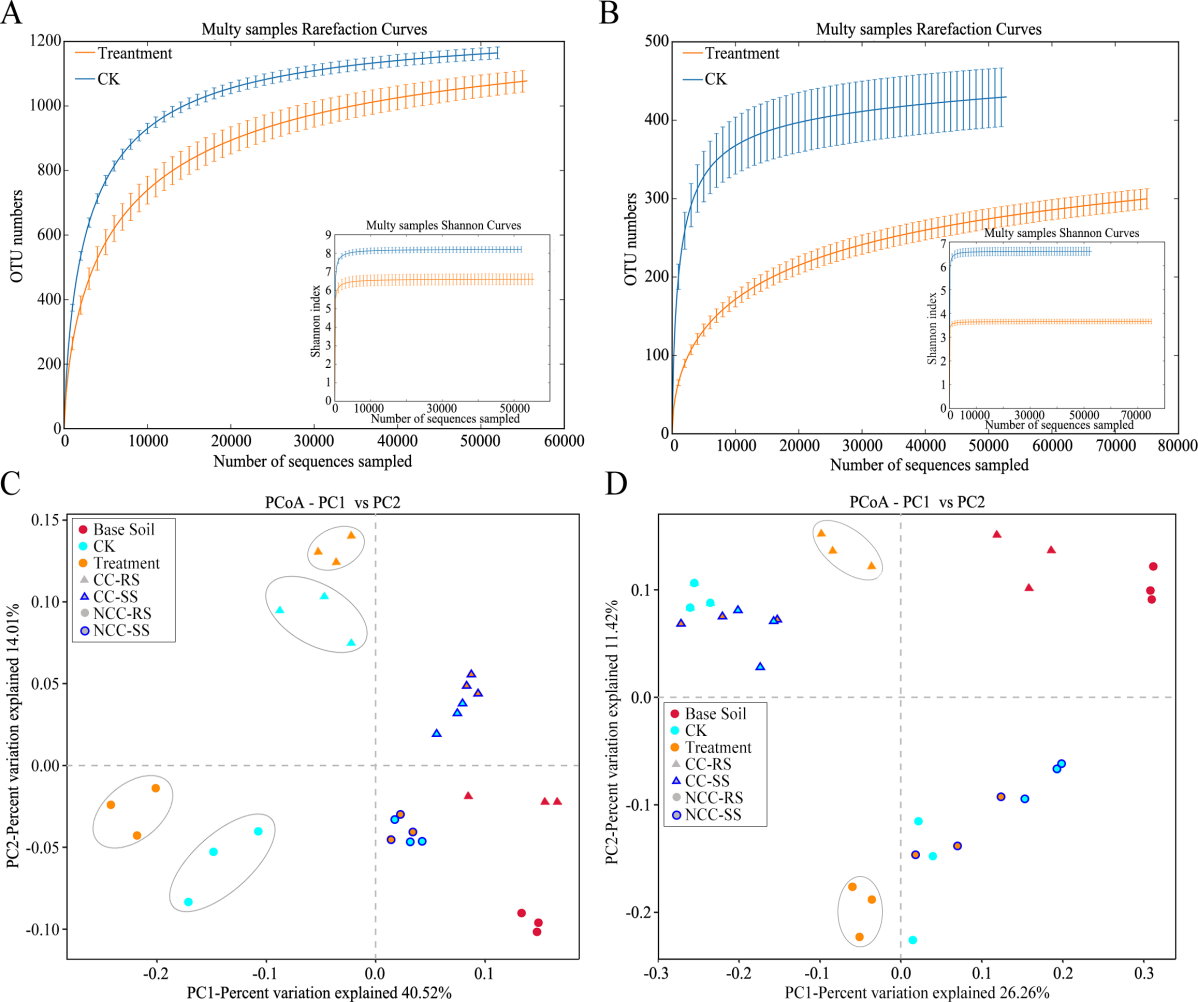
Diversity of microbial communities influenced by microbial inoculants and continuous monocropping. (**A, B**) Rarefaction curves and Shannon’s index for alpha-diversity measures of OTUs comparing bacteria and fungi, respectively. Error bars correspond to one standard deviation out from the average of biological replicates. (**C, D**) Unconstrained PCoA (for principal coordinates PCo1 and PCo2) with weighted unifrac distance showing root bacteria and fungi separated in the first two axes (*P* < 0.001, PERMANOVA).

Measurement of within-sample diversity (alpha diversity) revealed significant differences between samples in continuous and non-continuous cropping, as well as between microbial inoculant treatment and CK (*t*-test, *P* < 0.05, Supplementary Table S2; Fig. S1). Rarefaction plots of OTUs and Shannon’s index (Fig. 2A) confirmed that the bacteria in microbial inoculant treatment samples was significantly less diverse than that of CK samples and exhibited lower species richness and diversity regarding community diversity. This indicated that the application of the microbial inoculant resulted in the recruitment of fewer bacterial species. Furthermore, the diversity and richness of fungal communities characterized by rarefaction plots of OTUs and Shannon’s index were significantly decreased in treatment groups (Fig. 2B), suggesting that the application of an inoculant of bacterial origin had a negative influence on soil fungal community diversity.

To elucidate the degree by which microbial communities were influenced by continuous monocropping, microbial inoculant treatment, and location in root regions, and which factor(s) could be the underlying driving forces of microbial community variation in the data, PCoA was conducted based on the weighted UniFrac distance matrix in combination with PERMANOVA (Fig. 2C and D). The plots revealed clear differences in the root bacteria of flue-cured tobacco, which formed distinct clusters for root regions of the rhizosphere and root-surrounding soil that separated along the first coordinate, explaining 40.52% of the variation. PERMANOVA of pairwise distances between bacterial communities indicated that the microbiota differed significantly (R^2^ = 0.28, *P* < 0.001). Continuous and non-continuous cropping samples separated along the second coordinate, which explained 14.01% of the variation. Although microbial inoculant treatment did not separate the samples along the first two principal components, divergences in the β-diversity were still identified within each cluster separated by treatment, and this observation was supported by the PERMANOVA statistic (R^2^ = 0.202, *P* = 0.003). As shown in Fig. 2C, treatment and CK groups showed relatively obvious separation in continuous cropping samples but were vaguely divided within clusters of non-continuous cropping samples. This suggested the effects of the microbial inoculant on bacterial community diversity were more pronounced in the continuous monocropping system. With regard to the fungal community, a clear separation was identified between continuous and non-continuous cropping samples, which separated along the first and second coordinates. Within each cluster, divergences were also affected by root regions and microbial inoculant treatment, which separated the rhizosphere soil samples with inoculant treatment from other samples (Fig. 2D). This suggested that the effects of the microbial inoculant on β-diversity of the fungal community were predominantly reflected in rhizosphere fungi between continuous and non-continuous monocropping systems.

### Prediction of microbiota biomarkers and interactions in microbial co-occurrence networks

**Fig 3:**
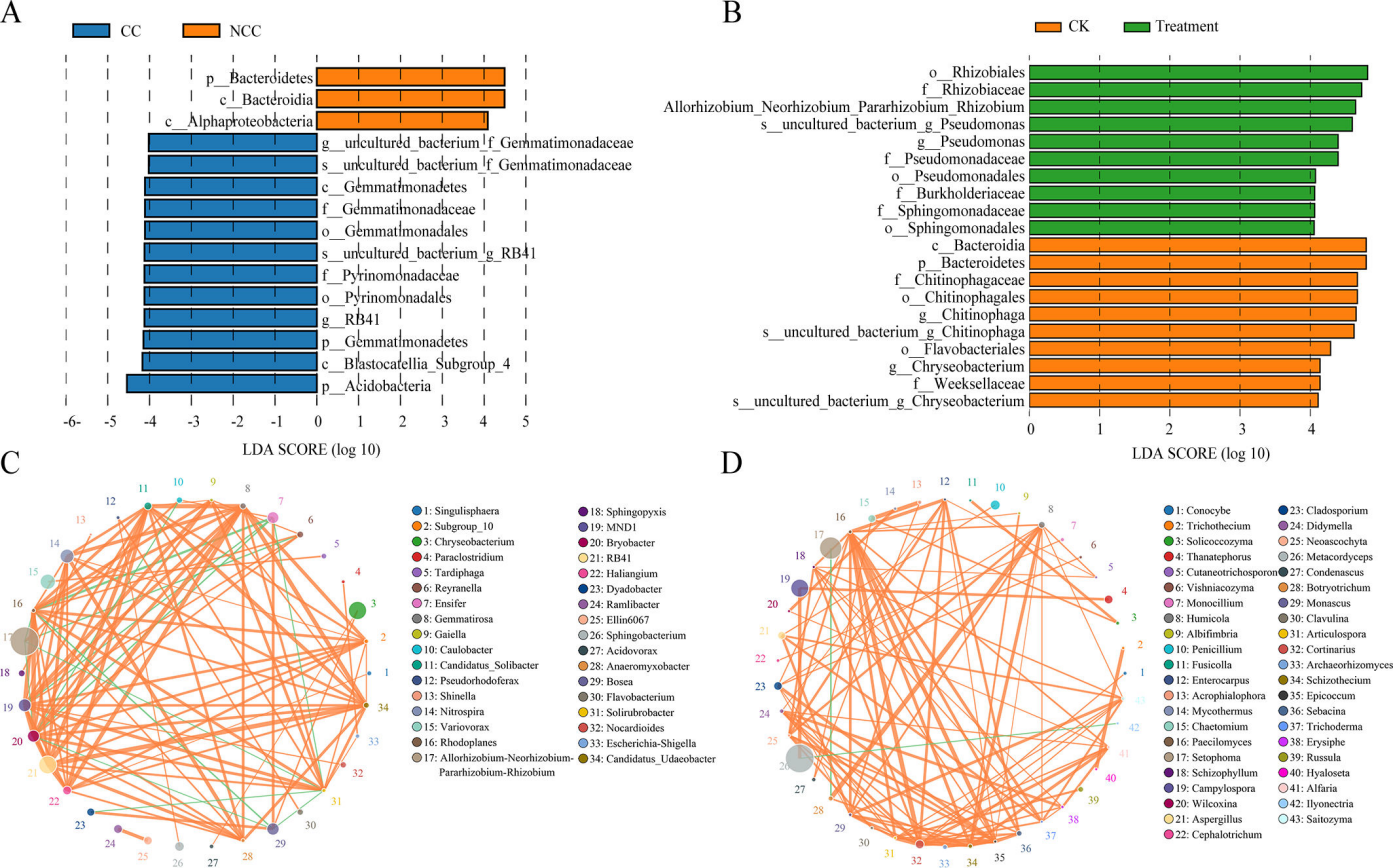
Graphics of linear discriminant analysis (LDA) effect size (LEfSe) of the biomarker prediction profiles between continuous and non-continuous monocropping samples (**A**) and between microbial inoculants treatment and CK groups (**B**). The threshold on the logarithmic LDA score for discriminative features was set to 4.0 at an FDR-adjusted *P*-value < 0.05. Taxa–taxa interactions at the species level in (**C**) bacterial and (**D**) fungal co-occurrence networks using Spearman’s rank correlation analysis set at a permutation value of 200, correlation >0.6, and *P*-value < 0.05. Circles represent species, and the size of the circle represents abundance. The edges represent the correlation between the two species, and the thickness of the edge represents the strength of the correlation. Orange lines represent a positive correlation and green lines represent a negative correlation.

The linear discriminant analysis (LDA) effect size (LEfSe) method was used to examine the differences in microbial communities between samples and identify bacterial biomarkers in each sample (capped at log_10_LDA score >4.0 and *P*-value < 0.05) (Fig. 3A and B). *Bacteroidetes* and *Alpha-proteobacteria* were defined as the featured biomarkers in non-continuous cropping soils, while *Acidobacteria* (including *Pyrinomonadaceae* in the phylum *Acidobacteria*) and *Gemmatimonadetes* were biomarkers in continuous cropping soils. For the biomarkers between treatment and CK groups, *Rhizobium*, *Pseudomonas*, *Sphingomonadaceae*, and *Burkholderiaceae* in the phylum *Proteobacteria* accumulated in a high relative abundance following application of the microbial inoculant, while the bacterial taxa biomarkers in the CK group were *Chitinophagaceae*, *Weeksellaceae*, and *Flavobacteriales* in the phylum *Bacteroidetes*.

Differences and interactions in bacterial and fungal co-occurrence patterns under continuous monocropping and microbial inoculant treatment were further revealed by co-occurrence network analysis at the genus level. To identify the common positive and negative interactions between microbiota, samples were combined and then correlations were calculated using Spearman’s rank correlation analysis, with a correlation value of >0.6 with 200 permutations at *P*-value < 0.05 (Table 4). The taxa networks of bacteria consisted of 34 nodes with 83 positive and 13 negative correlations (Fig. 3C). *Allorhizobium–Neorhizobium–Pararhizobium–Rhizobium* was predominant in the network, showing a positive correlation with *Caulobacter* and a negative correlation with several bacterial taxa, such as *Reyranella*, *Bryobacter*, *Rhodoplanes*, and *Solirubrobacter. Bryobacter* and *Rhodoplanes* were positively correlated with each other (R^2^ = 0.9693). Another hub node that was relatively abundant was *Chryseobacterium*, which showed a positive correlation with *Acidovorax* and *Bosea*. For the fungal network, which showed higher genera richness than the bacterial network, 43 nodes with two negative and 83 positive correlations were identified (Fig. 3D). *Metacordyceps* was enriched and exhibited a positive correlation with the enriched fungal genus *Campylospora*, but showed a negative correlation with *Ilvonectria*. Moreover, a negative correlation was identified between *Setophoma* and *Botryotrichum*, in which *Setophoma* was relatively abundant but exhibited a unique antagonistic correlation in the fungal network.

**TABLE 4:** Topological properties of communities in bacterial and fungal networks

Network properties	Bacterial network	Fungal network
Number of nodes	34	43
Number of edges	96	85
Modularity	0.127	0.126
Network density	0.597	0.619
Average shortest path length	1.406	1.398
Average clustering coefficient	0.768	0.8

### Metabolomics analysis of the non-targeted soil metabolites

**Fig 4:**
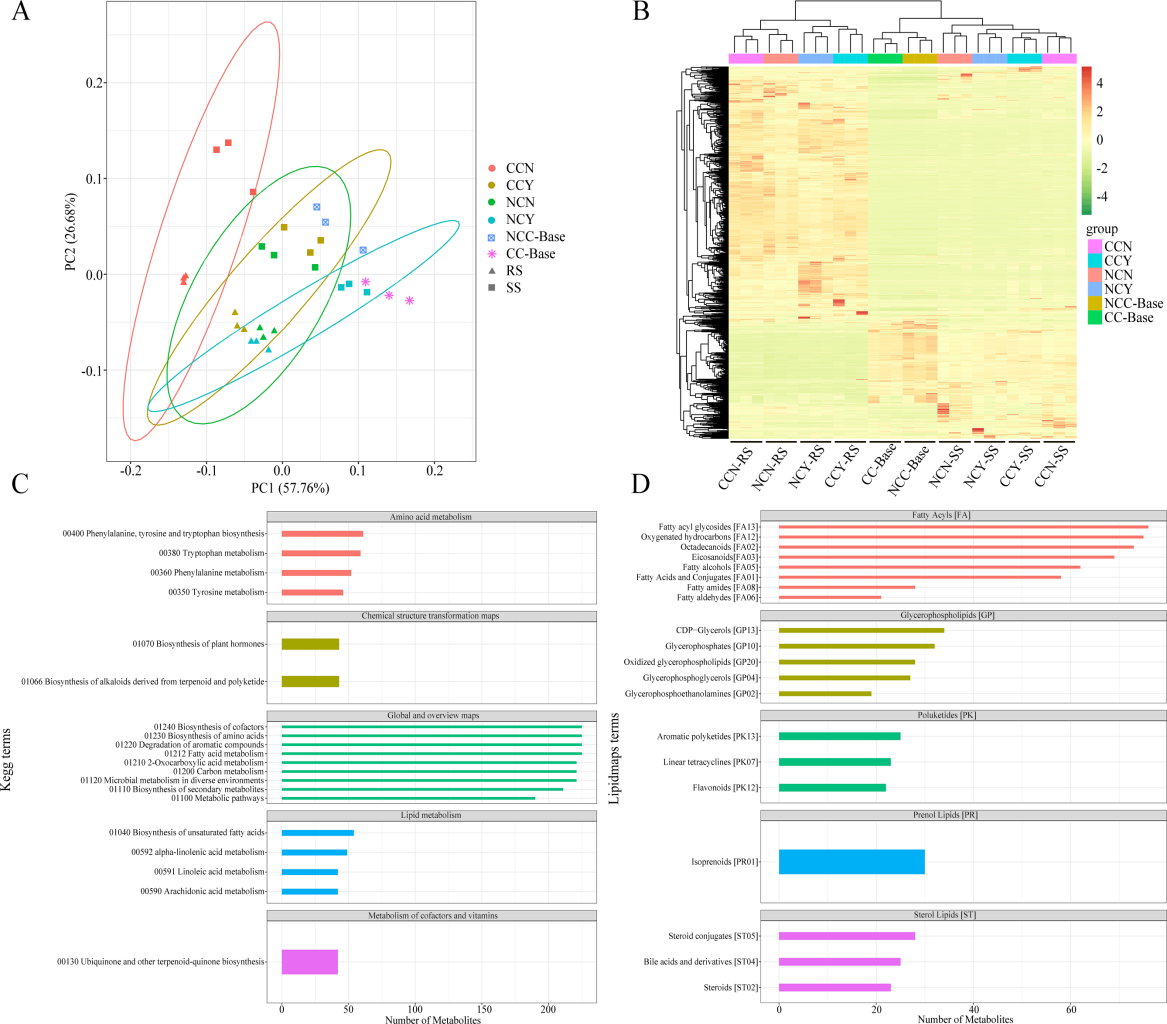
Metabolomic analysis of soil treated with microbial inoculants. (**A**) Principal component analysis (PCA) of metabolite profiles among 30 samples. (**B**) HCA for the metabolites based on their normalized abundances. (**C, D**) Annotated metabolites in the KEGG database (**C**) and LIPID MAPS database (**D**).

To determine the response of soil metabolic activities to the microbial inoculant, 30 soil samples were analyzed using metabolomics on the UPLC-MS/MS platform. A total of 2,485 soil metabolites were detected based on LC-MS analyses in this study, including 1,290 detected by positive ion mode (Table S3) and 1,195 by negative ion mode (Table S4). PCA was used to analyze differences among treatment samples. Score plots based on the first two components showed that the groups of soils in microbial inoculant treatment or not were well separated in continuous cropping and non-continuous cropping samples, respectively (Fig. 4A). In addition, hierarchical clustering analysis (HCA) of all samples (Fig. 4B) further demonstrated that the metabolite clustering was different between rhizosphere and root-surrounding soils, as well as between inoculant treatment and CK in rhizosphere soils. To understand the functional characteristics and classifications of metabolites, the identified metabolites were annotated using the KEGG database. This annotation revealed that the metabolites were predominantly involved in amino acid metabolism, lipid metabolism, biosynthesis of plant hormones and alkaloids, degradation of aromatic compounds, and microbial metabolism in diverse environments (Fig. 4C). To explore the relationships between these metabolites and lipids, the LIPID MAPS database was used to annotate the metabolites. There were 885 metabolites related to fatty acyls, glycerolipids and glycerophospholipids, polyketides, prenol lipids, and sphingolipids (Fig. 4D). Moreover, 22 and 30 metabolites were related to flavonoids and isoprenoids, respectively.

**Fig 5:**
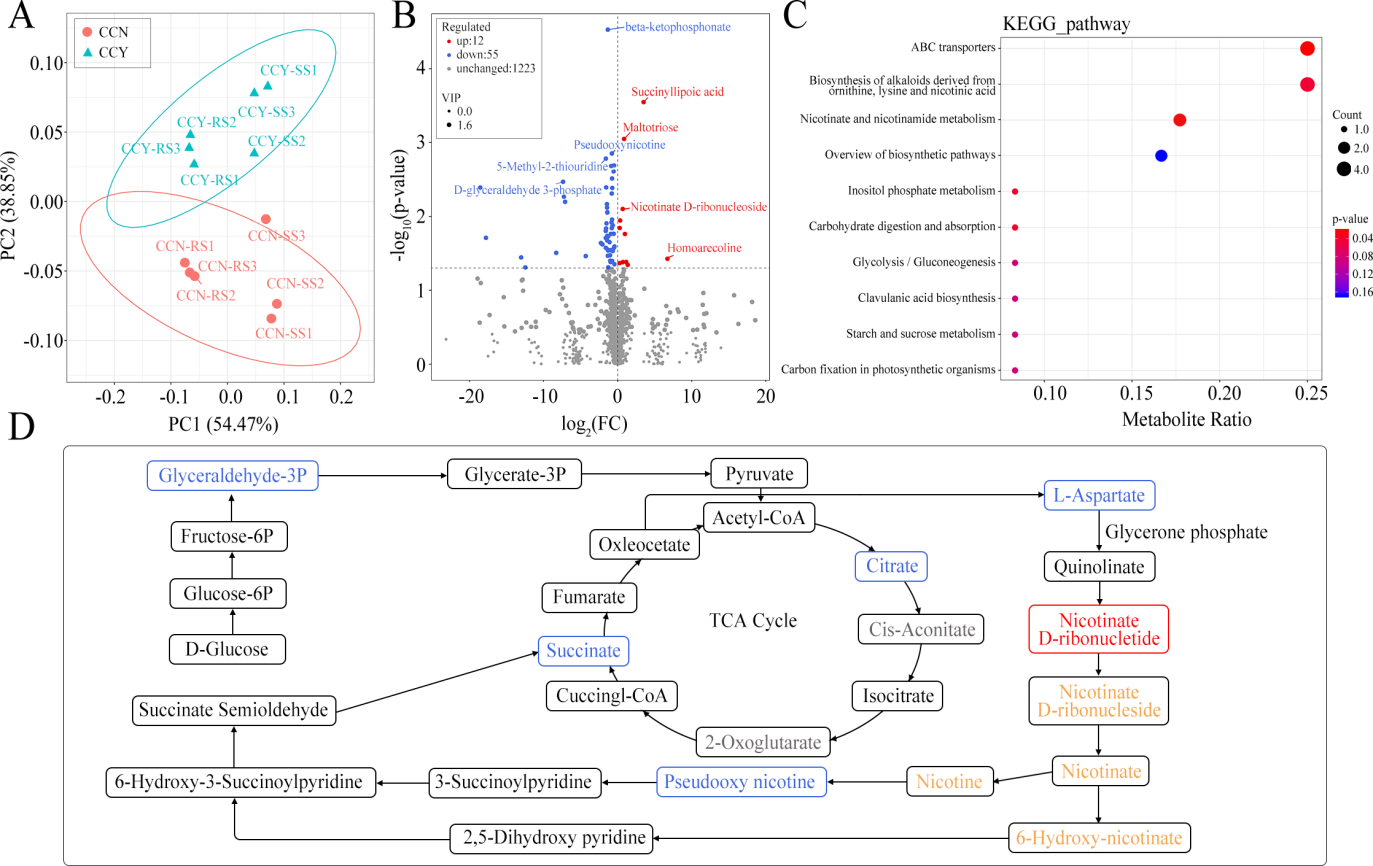
Differential metabolomic analysis of tobacco rhizosphere soils between treatment and CK groups in continuous cropping samples. (**A**) Principal component analysis (PCA) of metabolite profiles. (**B**) Volcano map of differential metabolites exhibiting upregulated and downregulated expression. Blue and red dots represent downregulated and upregulated metabolites, (*P*-value < 0.05), respectively; gray dots represent non-differential metabolites. (**C**) Scatter plot of top 10 KEGG pathways enrichment for differential metabolites. (**D**) Metabolic pathway map of differential marker metabolites in the synthesis and degradation of nicotine. Metabolites enclosed in a red or blue box indicate those that are significantly upregulated and downregulated, respectively, in inoculant-treated groups, and metabolites enclosed in an orange box indicate those that are upregulated but without significant difference.

Multivariate statistical methods were used to analyze the high degree of inter-group correlation. The differences within and between the treatment and CK groups in continuous cropping and non-continuous cropping samples were, respectively, analyzed using the OPLS-DA statistical model, to identify the key compounds responsible for the differentiation and obtain the impacts of the microbial inoculant on soil metabolites. The OPLS-DA score plots showed clear discrimination between the treatment and CK groups (Fig. S2 and S3). The OPLS-DA model indicated that the model could be used to screen for metabolites (R^2^Y = 0.994, Q^2^Y = 0.864 in continuous cropping; and R^2^Y = 0.966, Q^2^Y = 0.768 in non-continuous cropping) that differed among treatment and CK groups. Despite the identification of a large number of metabolites, only 67 were differentially abundant between treatment and CK groups in continuous monocropping, including 12 upregulated and 55 downregulated metabolites with inoculant treatment (Table S5). Similarly, in non-continuous cropping samples, there were 22 upregulated metabolites and 37 downregulated metabolites in the treatment group compared with the CK group (Table S6). These differentially abundant metabolites displayed the grouped discrimination by clustering (Fig. 5A; Fig. S4). Furthermore, to identify the compounds responsible for the difference, the VIP values in the OPLS-DA model combined with the fold changes (log_2_FC) and the *P*-values in the *t*-test were used to select variables with the most significant contribution to the discrimination of the compounds of two groups. Statistically significant differences were observed in the volcano map (Fig. 5B). In continuous monocropping, the contents of succinyllipoic acid, maltotriose, nicotinate D-ribonucleoside, and homoarecoline were significantly increased in the treatment group while beta-ketophosphonate, pseudooxynicotine, 5-methyl-2-thiouridine, and D-glyceraldehyde 3-phosphate were markedly reduced (VIP >2.5, *P* < 0.05). Through KEGG pathway annotation and functional enrichment, 23 metabolites of these 67 distinct metabolites were mapped onto 18 different KEGG metabolic pathways, which were involved in the overview of the biosynthetic pathway, starch and sucrose metabolism, flavone and flavonol biosynthesis, terpenoid backbone biosynthesis, clavulanic acid biosynthesis, etc. However, the KEGG-enrichment scatterplot showed that only five distinguishing pathways were significantly enriched in the treatment group compared with the CK group, including ABC transporters; biosynthesis of alkaloids derived from ornithine, lysine, and nicotinic acid; nicotinate and nicotinamide metabolism; inositol phosphate metabolism; and carbohydrate digestion and absorption (*P* < 0.05; Fig. 5C).

A metabolic pathway involving 12 differential metabolites between treatment and CK groups under continuous monocropping was generated by searching the KEGG pathway database (Fig. 5D). This pathway was involved in the synthesis and degradation of nicotine and included the significantly different metabolites of D-glyceraldehyde 3-phosphate, citrate, succinate, L-aspartate, and nicotinate D-ribonucleotide in nicotine synthesis, and pseudooxynicotine in nicotine degradation. Microbial inoculant treatment significantly upregulated the content of nicotinate D-ribonucleotide (involved in nicotine synthesis), resulting in the relative increases of nicotinate D-ribonucleoside, nicotinate, nicotine, and 6-hydroxynicotinate in the same pathway (although not significant). In the nicotine degradation pathway, the key compound pseudooxynicotine, which is catalyzed by nicotine dehydrogenase and then further demethylated to form 2,5-dihydroxy-pyridine and succinate, was significantly decreased in content and resulted in the reduction of the final product succinate. These results indicated that the application of the microbial inoculant indirectly affected nicotine accumulation and nicotinate metabolism in rhizosphere soils of tobacco.

### Correlation between the relative abundances of microorganisms and metabolites

**Fig 6:**
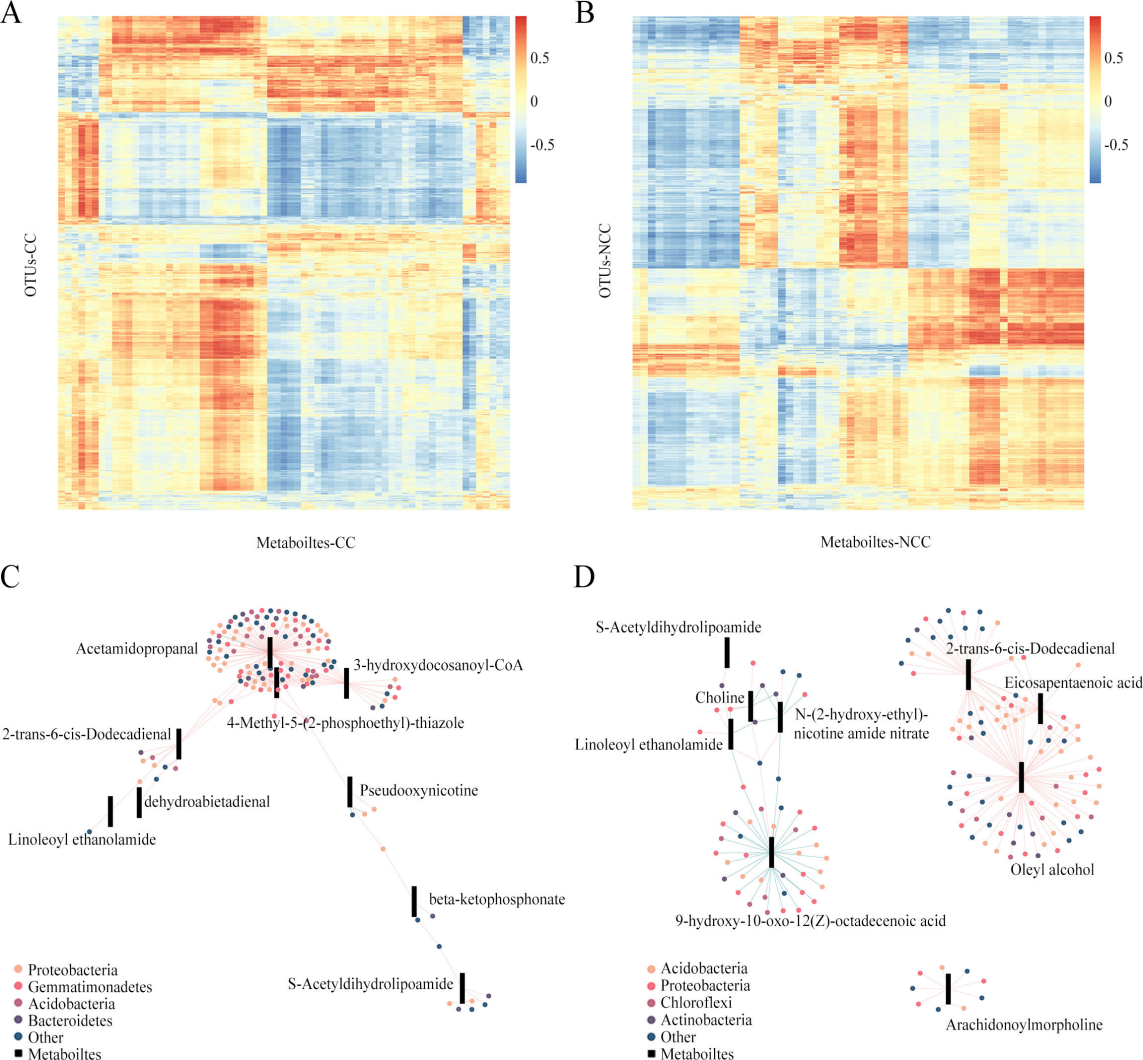
Correlation of microorganisms and metabolites between treatment and CK groups in continuous and non-continuous cropping samples. (**A, B**) Heatmaps in Pearson correlation analysis. The vertical axis represents normalized bacterial OTUs, and the horizontal axis represents the differential metabolites. (**C, D**) Correlation network analysis. The circles represent bacterial OTUs and colors represent different phyla; rectangles represent different metabolites, and red and blue lines represent positive and negative correlations, respectively.

To elucidate the relationship between microbes and their metabolites in rhizosphere soils, a correlation analysis was performed between microorganisms at the phyla level and metabolites based on abundance. Significant (*P* < 0.05) correlations were observed linking the significantly different bacteria with differential metabolites between treatment and CK groups in continuous and non-continuous cropping systems, respectively (Fig. 6A and B). To identify candidate metabolites that could influence the bacterial and fungal communities, weighted co-expression network analysis (WGCNA) was performed on the metabolites, in which the metabolites are divided into different modules. The metabolites in each module were then combined with the above correlation analysis results to obtain the correlation between metabolites and OTUs in each module. In the network under the continuous cropping system (Fig. 6C), acetamidopropanal, 4-methyl-5-(2-phosphoethyl)-thiazole, and 3-hydroxydocosanoyl-CoA were clustered together through significantly positive correlations with 108, 39, and 23 bacteria, respectively, belonging to the phyla *Proteobacteria*, *Gemmatimonadetes*, *Acidobacteria*, and *Bacteroidetes*. Among these bacteria, *Gemmatimonadaceae* bacteria (OTU464), *Anaeromyxobacter* sp. (OTU566), and *Burkholderiaceae* bacteria (OTU7620) were positively correlated with 2-trans-6-cis-dodecadienal, which was clustered with linoleoyl ethanolamide and dehydroabietadienal through positive correlations with *Rhodanobacter* sp. (OTU995) and *Ktedonobacteria* (OTU2044). In addition, *Acidobacteria* bacterium WY67 (OTU628) showed a positive relationship with acetamidopropanal, 4-methyl-5-(2-phosphoethyl)-thiazole, and pseudooxynicotine. The metabolite pseudooxynicotine was negatively correlated with *Micromonosporaceae* (*Actinobacteria*) and *Phyllobacterium* sp. (*Rhizobiaceae*, *Proteobacteria*). Pseudooxynicotine and beta-ketophosphonate were both positively correlated with *Sphingobium* sp. (OTU25). Beta-ketophosphonate, showing a negative correlation with *Streptomyces scabrisporus* and a positive correlation with *Sphingobacterium* sp., was clustered with S-acetyldihydrolipoamide through a positive correlation with *Lachnospiraceae* bacterium (OTU782).

Under the non-continuous cropping system, the correlation network presented as three sub-networks (Fig. 6D). The first one consisted of three metabolites—oleyl alcohol, eicosapentaenoic acid, and 2-trans-6-cis-dodecadienal—showing positive correlations with 72, 23, and 37 bacteria, respectively, which belonged to the phyla *Acidobacteria*, *Proteobacteria*, *Chloroflexi*, and *Actinobacteria*. Nearly 30 bacteria were shared by two or three metabolites. The second sub-network only showed one metabolite—arachidonoylmorpholine—positively correlated with nine bacteria. The third sub-network predominantly presented negative correlations between metabolites and bacteria. The metabolite 9-hydroxy-10-oxo-12(Z)-octadecenoic acid had highly negative correlations with 35 bacteria and was positively correlated with only one bacterium, *Chitinophaga* (OTU1192), which was also positively correlated with linoleoyl ethanolamide, N-(2-hydroxy-ethyl)-nicotine amide nitrate, and choline. In addition, the above four metabolites were clustered with each other through negative correlations with the respective bacteria *Caulobacteraceae* bacterium (OTU1892), *Hymenobacter* sp. (OTU1623), *Burkholderiaceae* bacterium (OTU94), *Aeromicrobium* sp. (OTU442), *Acidimicrobiia* bacterium (OTU763), and *Microbacterium* sp. (OTU75).

## DISCUSSION

In this study, high-throughput sequencing and UHPLC-MS/MS were used to evaluate whether microbial inoculant treatment resulted in differences in bacterial and fungal communities and metabolomics in tobacco rhizosphere soil based on the improvement of tobacco yield, disease resistance, and intrinsic quality. Soil microorganisms, especially rhizosphere microorganisms, form complex microbial communities that regulate nutrient cycling by releasing a variety of soil enzymes, which affect soil properties, plant growth, and ecosystem sustainability (28, 29). Conversely, microbial communities are affected by fertilizers and other agricultural interventions that impact plant root growth. Bacteria are the most abundant and diverse types of soil microorganisms and play an important role in agricultural ecosystems by participating in the synthesis of humus, mineralization and degradation of organic matter, promotion or inhibition of plant growth, and nutrient cycling (30). Our results demonstrated that the application of a microbial inoculant altered the diversity of bacterial communities in tobacco rhizosphere soils under continuous monocropping, such as increasing the relative abundance of the phyla *Proteobacteria*, *Cyanobacteria*, and *Bacteroidetes*, while decreasing the number of *Acidobacteria*, *Gemmatimonadetes*, etc. This may be due to changes in the environmental conditions for the growth of rhizosphere *Acidobacteria* or *Gemmatimonadetes* or could be due to a decrease in competition from other microbes (31), which may also affect metabolism in plants and soil. Simultaneously, our results proved that the application of microbial inoculant had a positive effect on the bacterial structure in the tobacco rhizosphere. As reported in previous studies, the healthy rhizosphere is a hotspot of *Bacteroidetes* and *Cyanobacteria* genes, providing functional capacity for the transformation of labile and recalcitrant organic C, N, P, and S compounds (32). In addition, the beta-diversity results confirmed the significant differences in bacterial and fungal communities between the tobacco rhizosphere and root-surrounding soils, indicating that the selectivity of root to rhizosphere microbes is an important factor leading to the differences in microbial community structure in the rhizosphere compared with the root-surrounding or bulk soils (33). That is, shifts in bacterial and fungal communities in response to the rhizosphere are also associated with changes in root exudation patterns (34).

Prediction of biomarkers in the tobacco rhizosphere by LEfSe analysis (log_10_LDA score >4.0 and *P*-value < 0.05) identified *Rhizobium*, *Pseudomonas*, and *Sphingomonas*, which belong to the phylum *Proteobacteria*, as biomarkers of the microbial inoculant treatment group, and these bacteria have been widely documented to promote plant growth. *Pseudomonas* and *Rhizobiaceae* are nitrogen-fixing bacteria (35, 36). *Pseudomonas* was reported to attach to roots and efficiently colonize root surfaces, while *Rhizobiaceae* are symbiotic with plant hosts for nitrogen fixation. These bacteria have the potential to promote sustainable plant growth (1, 37). It has also been reported that these bacteria can produce gibberellin acid (GA) and indole-3-acetic acid (IAA) (38), can induce root growth, and are positively correlated with soil carbon and nitrogen content, suggesting that these bacteria are also responsible for the improvement of tobacco leaf quality observed in this study.

Metabolome analysis revealed significant differences in microbial composition as well as metabolites of rhizosphere and root-surrounding soils. Soil metabolites mainly originate from plant roots and microorganisms. The composition of root exudates varies with plant species, genotype, and environmental stress (39, 40). Changes in the composition and content of soil metabolites can reveal direct or indirect responses of soil microbes to soil nutrients (41). The change in microbial species and abundance determines the change in soil metabolites to a certain extent and then determines the metabolism cycle of exogenous nutrients in soil. Therefore, it is of great significance to reveal the correlation between soil metabolism and bacterial community (31). The application of microbial inoculant significantly affected the metabolite profile of the rhizosphere soil; the contents of succinyllipoic acid, maltotriose, nicotinate D-ribonucleoside, and homoarecoline were significantly increased, and some metabolic pathways were disrupted. The pathways of nicotinate and nicotinamide metabolism and the biosynthesis of alkaloids derived from ornithine, lysine, and nicotinic acid are of particular interest because they involve the synthesis and degradation of the important compound nicotine in tobacco.

Nicotine is a major secondary metabolite of tobacco plants, and some nicotine is released into the soil environment through root exudates and residual root decomposition (42). From a positive perspective, nicotine released from the rhizosphere enhances the absorption of nitrogen, calcium, iron, and zinc, thereby promoting seedling emergence and vitality, chlorophyll content, and subsequent crop growth. Passive release of nicotine from the root zone of the meristem to the soil rhizosphere is instrumental in protecting plants from pathogenic soil bacteria and fungi, thereby reducing competition for soil nutrients that may be metabolized by these pathogens (43). In addition, nicotine is closely related to nitrogen levels and follows a similar accumulation pattern. High levels of nutrient nitrogen can increase the content of nicotine and nitrate in tobacco leaves (44). Therefore, after being released through the tobacco rhizosphere, nicotine promotes the absorption of nitrogen by the roots. This study found that the tobacco dry weight, nicotine content, and nitrogen content in microbial inoculant treatment groups were higher than those of the control groups. Moreover, in terms of rhizosphere metabolic pathways, the contents of metabolites were increased in the nicotine synthesis pathway but decreased in the nicotine degradation pathway—that is, nicotine content was accumulated in the rhizosphere soil—following the application of the microbial inoculant. This indicated that the inoculant treatment promotes nutrient balance in the soil by influencing the structure and activity of rhizosphere microbial communities and participating in nitrogen metabolism pathways (45). An alternative explanation is that the inoculant treatment increases the total nitrogen in the soil owing to the inhibitory effect of nicotine on soil bacteria involved in converting ammonia into nitrate, for example, *Nitrosomonas*, *Nitrococcus*, and *Nitrobacter* (46). This process also helps to minimize soil nitrogen loss (47). In addition, soil nitrogen metabolism directly or indirectly interacts with tobacco roots, triggering a series of reactions among root systems and affecting the production of secondary metabolites, which ultimately affects the quality of tobacco leaves (48).

However, studies have also demonstrated that nicotine can negatively affect the proliferation of some beneficial bacteria and fungi and the availability of potassium and phosphorus. Only microorganisms tolerant to high concentrations of nicotine can persist in the root zone, thus reducing competition for soil nutrients (43). One such example is *Pseudomonas*, which is highly tolerant to nicotine toxicity in the pH range of 6.5–7.0 (49). *Pseudomonas* sp. strains CS3, Nic22, and ZUTSKD could tolerate nicotine concentrations in soil up to 5 g L^−1^ and could degrade nicotine efficiently in soils at 30–34°C and pH 6.0–7.0 (49, 50). Strain HF-1, which was also identified as a member of the genus *Pseudomonas*, had a higher nicotine degradation efficiency of 99.6% in soil at pH 6.5–7.5 (51). Thus, nicotine adsorbed in soil colloids has residual effects on plant growth, as well as the survival and proliferation of beneficial soil bacteria and fungi. With increasing years of tobacco continuous cropping, nicotine residues in soil increased total soluble phenolic acids, which may negatively impact tobacco crops and beneficial soil bacteria and fungi (4). There are ongoing studies investigating the allelopathic effects of continuous tobacco cropping on tobacco root colonization and growth and on the chemicals in rhizosphere soils based on research findings on other important crops (52, 53). For example, continuous cropping of poplar increased the accumulation of phenolic acids and decreased ammoniated substances in the soil; and in the rhizosphere of continuous cropping soybean, the accumulation of phenolic acids was also observed (54). Microorganisms using phenolic acids as carbon sources play a key role in autotoxicity in tobacco continuous cropping soils; consequently, the effect of fertilizers on the functional diversity of microorganisms is a research hotspot in eliminating the soil autotoxicity of continuous cropping tobacco. In this study, the contents of p-coumaric acid, ferulic acid, ethyl salicylate (a hydroxybenzoic acid), phthalic acid mono-2-ethylhexyl ester, and other phenolic acids were significantly decreased in inoculant treatment groups. This indicated that phenolic acid-dependent rhizosphere microorganisms were stimulated by the inoculant treatment, leading to the consumption of phenolic acids in the soil. Studies have shown that phenolic acids have the potential to stimulate the growth of specific soil microbial species such as *Burkholderia* spp. and contribute to increased populations of *Burkholderia* colonizing the rhizosphere (55). *Burkholderia* is known for its impressive ability to catabolize aromatic compounds and is the major bacterial degrader of aromatic compounds in soil (56). Our study corroborated this claim, as *Burkholderia* was identified as a biomarker in the inoculant-treated group. However, it should be noted that metabolomics analysis is the analysis of biological metabolites at a specific time under specific conditions (57), and the dynamic changes of soil metabolite profiles and their correlation with soil microbial community structure need to be further studied.

## Data Availability

The raw sequencing data generated in this study have been deposited in the NCBI Sequence Read Archive (SRA) under accession number PRJNA1014696 for microbiome analysis and National Genomics Data Center (NGDC) under accession number PRJCA026132 for metabolome analysis.

## SUPPLEMENTAL MATERIAL

Fig. S1 spectrum.04046-23-s0001.pdf Rarefaction curves and Shannon's index for alpha diversity measures of OTUs comparing bacteria and fungus between continuous and non-continuous monocropping. Error bars correspond to one standard deviation out from the average of biological replicates.

Fig. S2 spectrum.04046-23-s0002.pdf Orthogonal partial least-squares discriminant analysis (OPLS-DA) of positive metabolites and cross-validation plot of OPLS-DA model between inoculant treated and CK groups in continuous (A~B) and non-continuous (C~D) monocropping, respectively.

Fig. S3 spectrum.04046-23-s0003.pdf Orthogonal partial least-squares discriminant analysis (OPLS-DA) of negative metabolites and cross-validation plot of OPLS-DA model between inoculant treated and CK groups in continuous monocropping.

Fig. S4 spectrum.04046-23-s0004.pdf Differential metabolomic analysis of tobacco rhizosphere soils between treatment and CK groups in non-continuous cropping samples.

Table S1 spectrum.04046-23-s0005.xlsx Effect of microbial inoculants on growth parameters and resistance of tobacco in harvest period.

Table S2 spectrum.04046-23-s0006.xlsx Alpha diversity indexes based on bacterial OTU level.

Table S3 spectrum.04046-23-s0007.xlsx Soil metabolites detected by positive ion mode.

Table S4 spectrum.04046-23-s0008.xlsx Soil metabolites detected by negative ion mode.

Table S5 spectrum.04046-23-s0009.xlsx Differentially abundant metabolites between inoculant treated and CK groups in continuous monocropping.

Table S6 spectrum.04046-23-s0010.xlsx Differentially abundant metabolites between inoculant treated and CK groups in non-continuous monocropping.

ASM does not own the copyrights to Supplemental Material that may be linked to, or accessed through, an article. The authors have granted ASM a non-exclusive, world-wide license to publish the Supplemental Material files. Please contact the corresponding author directly for reuse.
