# Supplementary material for: Integrative analysis of microbiome and metabolome revealed the effect of microbial inoculant on microbial community diversity and function in rhizospheric soil under tobacco monoculture
Source: Microbiol Spectr. 2024 Jul 11;12(8):e04046-23. doi: 10.1128/spectrum.04046-23 (PMC11302352; doi:10.1128/spectrum.04046-23)
Supplement: Fig. S1 — Rarefaction curves and Shannon's index for alpha diversity measures of OTUs comparing bacteria and fungus between continuous and non-continuous monocropping. Error bars correspond to one standard deviation out from the average of biological replicates. [file spectrum.04046-23-s0001.pdf]

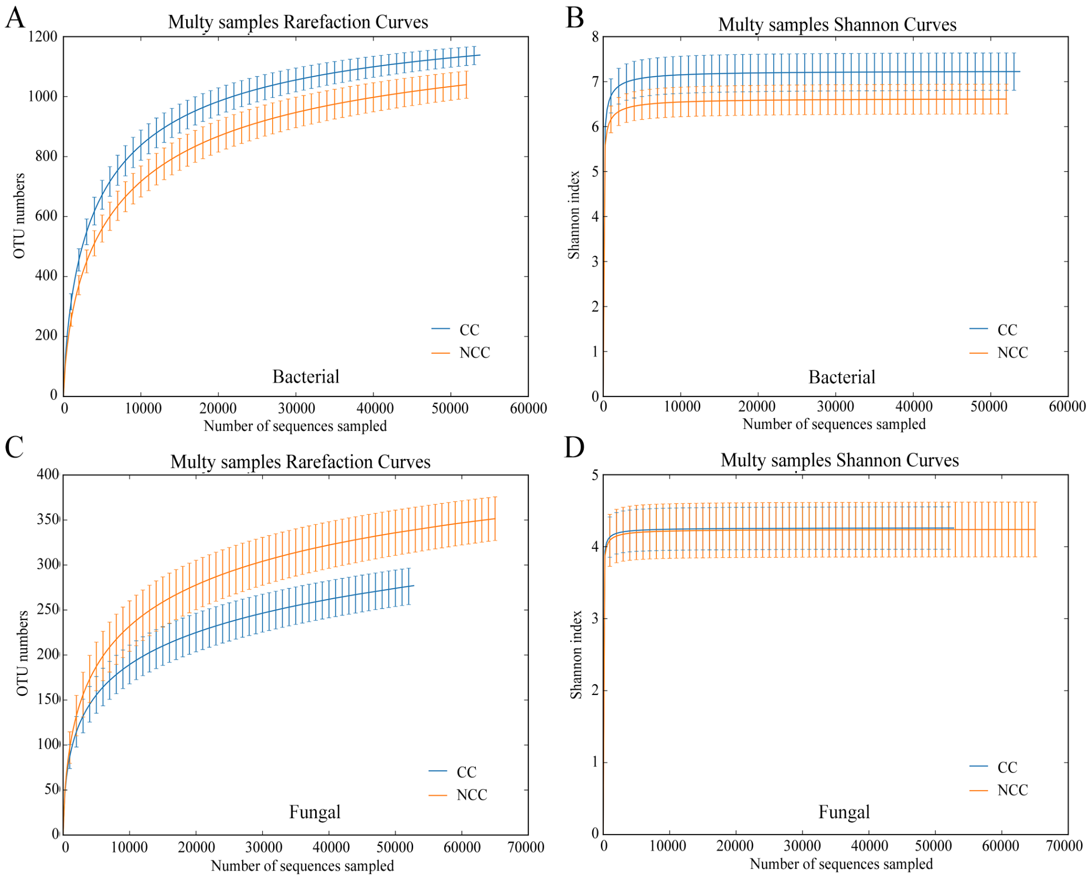

SUPPLEMENTARY FIGURE S1 | Rarefaction curves and Shannon's index for alpha diversity measures of OTUs comparing bacteria and fungus between continuous and non-continuous monocropping. Error bars correspond to one standard deviation out from the average of biological replicates.
