# Supplementary material for: Integrative analysis of microbiome and metabolome revealed the effect of microbial inoculant on microbial community diversity and function in rhizospheric soil under tobacco monoculture
Source: Microbiol Spectr. 2024 Jul 11;12(8):e04046-23. doi: 10.1128/spectrum.04046-23 (PMC11302352; doi:10.1128/spectrum.04046-23)
Supplement: Fig. S2 — Orthogonal partial least-squares discriminant analysis (OPLS-DA) of positive metabolites and cross-validation plot of OPLS-DA model between inoculant treated and CK groups in continuous (A~B) and non-continuous (C~D) monocropping, respectively. [file spectrum.04046-23-s0002.pdf]

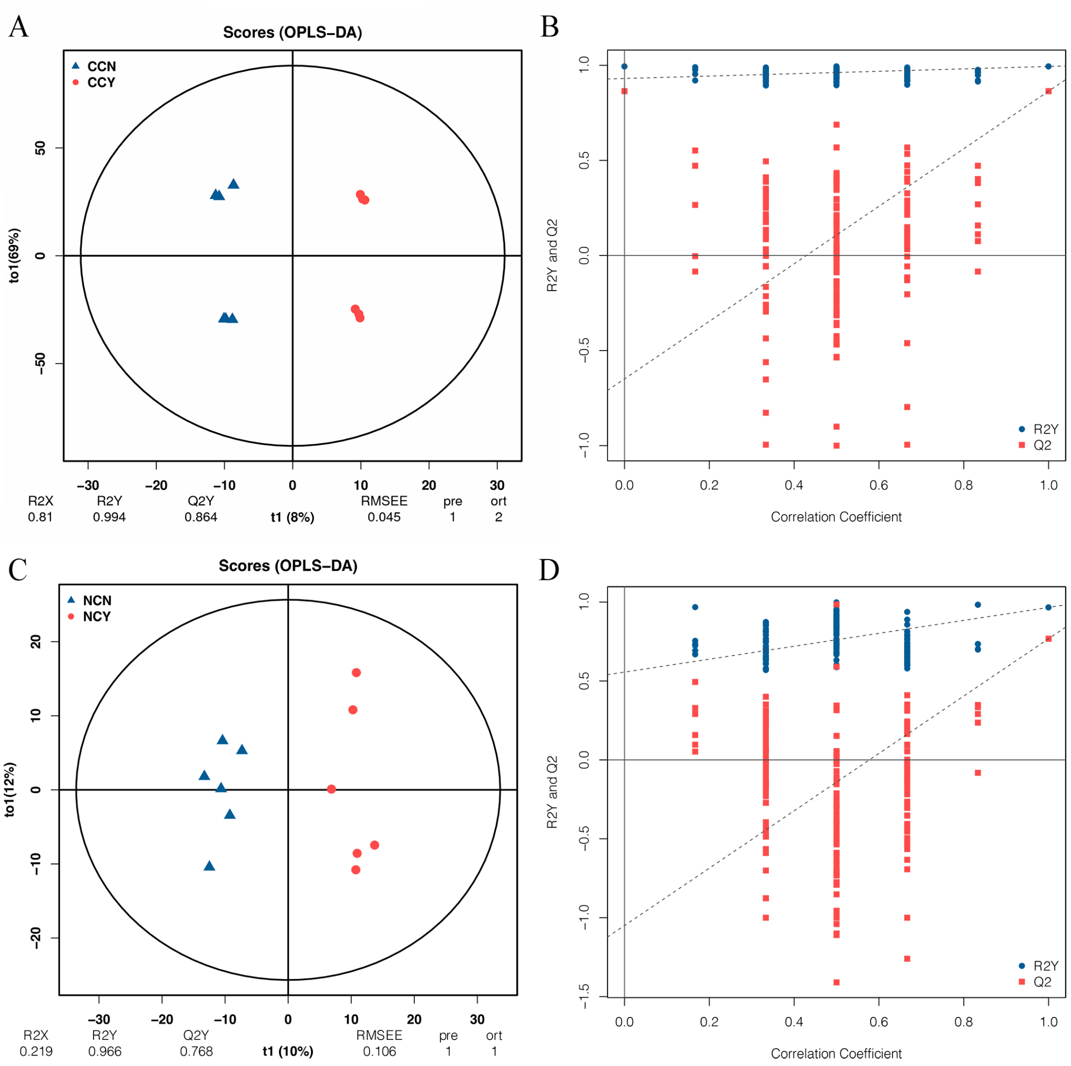

SUPPLEMENTARY FIGURE S2 | Orthogonal partial least-squares discriminant analysis (OPLS-DA) of positive metabolites and cross-validation plot of OPLS-DA model between inoculant treated and CK groups in continuous (A~B) and non-continuous (C~D) monocropping, respectively.
