# Supplementary material for: Integrative analysis of microbiome and metabolome revealed the effect of microbial inoculant on microbial community diversity and function in rhizospheric soil under tobacco monoculture
Source: Microbiol Spectr. 2024 Jul 11;12(8):e04046-23. doi: 10.1128/spectrum.04046-23 (PMC11302352; doi:10.1128/spectrum.04046-23)
Supplement: Fig. S3 — Orthogonal partial least-squares discriminant analysis (OPLS-DA) of negative metabolites and cross-validation plot of OPLS-DA model between inoculant treated and CK groups in continuous monocropping. [file spectrum.04046-23-s0003.pdf]

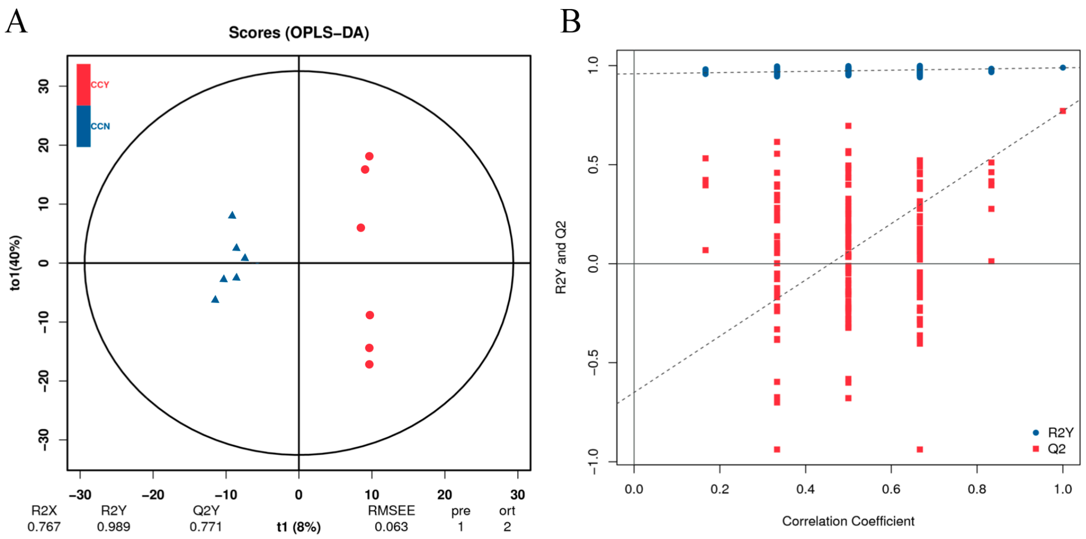

SUPPLEMENTARY FIGURE S3 | Orthogonal partial least-squares discriminant analysis (OPLS-DA) of negative metabolites and cross-validation plot of OPLS-DA model between inoculant treated and CK groups in continuous monocropping.
