# Supplementary material for: Integrative analysis of microbiome and metabolome revealed the effect of microbial inoculant on microbial community diversity and function in rhizospheric soil under tobacco monoculture
Source: Microbiol Spectr. 2024 Jul 11;12(8):e04046-23. doi: 10.1128/spectrum.04046-23 (PMC11302352; doi:10.1128/spectrum.04046-23)
Supplement: Fig. S4 — Differential metabolomic analysis of tobacco rhizosphere soils between treatment and CK groups in non-continuous cropping samples. [file spectrum.04046-23-s0004.pdf]

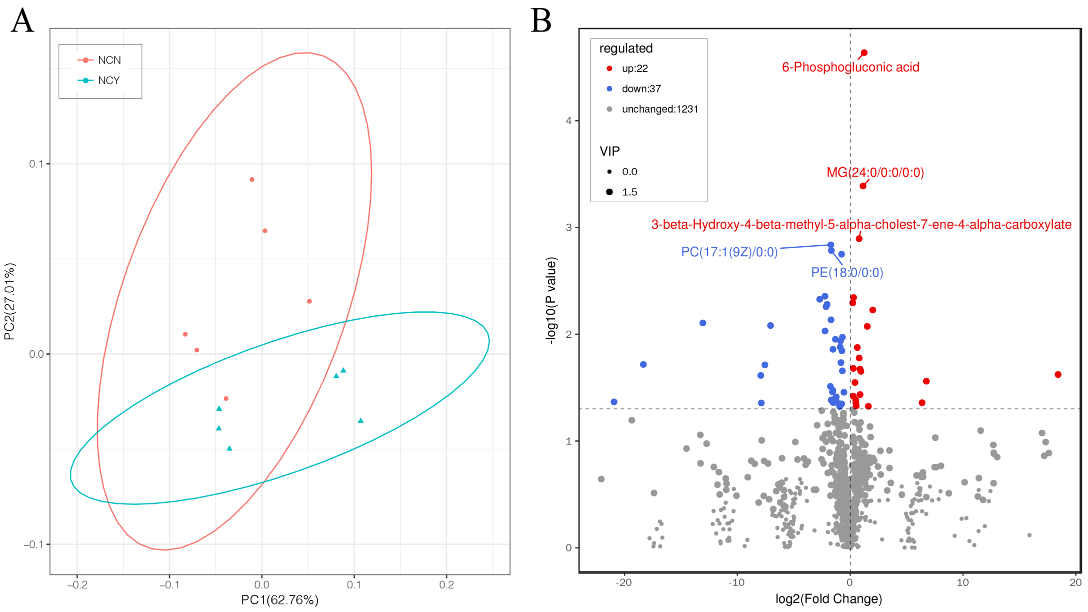

SUPPLEMENTARY FIGURE S4 | Differential metabolomic analysis of tobacco rhizosphere soils between treatment and CK groups in non-continuous cropping samples. (A) Principal component analysis (PCA) of metabolites profiles. (B) The expression volcano map of differential metabolites up and down regulate. Blue and red dots represented down- and up-regulated metabolites (*P*-value < 0.05);
